# Use of Genome Sequencing to Define Institutional Influenza Outbreaks, Toronto, Ontario, Canada, 2014–15

**DOI:** 10.3201/eid2403.171499

**Published:** 2018-03

**Authors:** Derek R. MacFadden, Allison McGeer, Taryn Athey, Stephen Perusini, Romy Olsha, Aimin Li, AliReza Eshaghi, Jonathan B. Gubbay, William P. Hanage

**Affiliations:** University of Toronto, Toronto, Ontario, Canada (D.R. MacFadden, A. McGeer, J.B. Gubbay);; Mount Sinai Hospital, Sinai Health System, Toronto (A. McGeer);; Public Health Ontario Laboratory, Toronto (T. Athey, S. Perusini, R. Olsha, A. Li, A. Eshaghi, J.B. Gubbay);; Harvard T.H. Chan School of Public Health, Harvard University, Boston, Massachusetts, USA (W.P. Hanage)

**Keywords:** influenza, genomic epidemiology, outbreaks, long-term care, hospital, institutional outbreak, whole-genome sequencing, public health, respiratory infections

## Abstract

Adequacy of the current clinical definition of institutional influenza outbreaks is unclear. We performed a retrospective genome sequencing and epidemiologic analysis of institutional influenza outbreaks that occurred during the 2014–15 influenza season in Toronto, Canada. We sequenced the 2 earliest submitted samples positive for influenza A(H3N2) from each of 38 reported institutional outbreaks in long-term care facilities. Genome sequencing showed most outbreak pairs identified by using the current clinical definition were highly related. Inclusion of surveillance samples demonstrated that outbreak sources were likely introductions from broader circulating lineages. Pairwise distance analysis using majority genome and hemagglutinin-specific genes enabled identification of thresholds for discrimination of within and between outbreak pairs; the area under the curve ranged 0.93–0.95. Routine genome sequencing for defining influenza outbreaks in long-term care facilities is unlikely to add significantly to the current clinical definition. Sequencing may prove most useful for investigating sources of outbreak introductions.

Current definitions for influenza outbreaks in hospitals or chronic/long-term care facilities (LTCFs) are ill-defined, being typically based on >2 symptomatic patients in a 48–72-hour period and >1 microbiologic sample documented as positive for influenza (*1*,*2*). However, this definition does not conclusively determine whether transmission events have occurred within the institution or if a linked outbreak is emerging. Influenza outbreaks in hospitals and LTCFs are associated with significant rates of illness and death (*3*). Annually, 1% of adults >65 years of age in North America are hospitalized because of influenza symptoms, and case-fatality rates have been reported at 50% in some groups (*4*). Outbreak prevention measures, including chemoprophylaxis and traditional infection control approaches, have demonstrated benefits in confirmed outbreaks (*5*). However, these benefits are balanced by resource expenditures, use of chemoprophylaxis in uninfected persons, and potentially detrimental interruptions in care introduced by infection prevention and control measures (*6*). The 2014–15 influenza season in North America was dominated by influenza A(H3N2) but had a poor vaccine match and efficacy to the circulating H3N2 strain, and high case numbers were counted (*7*). The US Centers for Disease Control and Prevention categorized the season as moderately severe and among the longest seasons in the previous decade (*8*).

By using currently employed direct fluorescent antibody or PCR diagnostic techniques, it is often impossible to determine whether detected influenza strains in an outbreak are related. It is probable that some outbreaks represent independently introduced cases, without direct transmission. Even in large institutional outbreaks of influenza, multiple strain types can be introduced (*9*). Although the purpose of identifying outbreaks and enacting infection control measures in facilities is to limit linked transmission (*1*), the identification of an outbreak may be more of a reflection of the force of infection in the population in general at that time: 25%–50% of LTCFs report >1 influenza outbreak annually (*4*). If facility outbreak rates mirror those of outbreaks in the general population, measures to limit transmission in the facility are expected to have limited benefit because cases are actually introduced from outside. Despite the aim of outbreak infection control measures to reduce institutional transmission, we have a limited understanding of the adequacy of the broadly applied clinical definition of influenza outbreaks created for identification of transmission. Genome sequencing has the potential to discern differences in influenza strains and clarify related and unrelated strains (*10*).

Genome sequencing is increasingly being incorporated into clinical care, including outbreak investigations and infection control (*11*–*13*). Recent technological advancements offer increasingly portable and rapid tools that have the potential to revolutionize clinical microbial diagnostics (*14*). However, to date, the role of genome sequencing in defining institutional influenza outbreaks has not been systematically evaluated. Here, we evaluate whether influenza genome sequencing could improve understanding of the utility of current influenza outbreak definitions and whether it could play a role in routine outbreak identification.

## Materials and Methods

We conducted a retrospective analysis of influenza outbreaks in LTCFs comprising chronic care hospitals, long-term care institutions, and retirement homes across the city of Toronto, Ontario, Canada, during the 2014–15 influenza season. We performed genome sequencing on 38 pairs of influenza-positive outbreak samples that had been collected and sampled prospectively throughout the outbreak season. Pairs of samples for each evaluated outbreak constituted the 2 earliest positive samples for influenza A(H3N2) from each outbreak, provided the outbreak had >2 adequate influenza-positive samples. Samples submitted from suspected outbreaks were initially screened by real-time reverse transcription PCR (RT-PCR). The Public Health Ontario research ethics board in the province of Ontario, Canada, approved this study.

### Influenza Season Epidemiology

We used prospectively collected data on influenza A(H3N2) outbreaks occurring in Toronto during the 2014–15 influenza season, which included the following variables: outbreak number, outbreak size, date of outbreak onset, and date of submitted samples. Publicly available information on weekly totals of PCR-confirmed influenza A–positive samples tested at Public Health Ontario Laboratory (PHOL), the reference microbiology laboratory for Ontario, were also included (*15*).

### Sequencing

We sequenced primary clinical specimens. For each sample, total nucleic acid was extracted on the easyMag extraction system (bioMérieux, Saint-Laurent, Quebec, Canada) by using 250 μL of sample with 25 μL eluate. We used universal primers (MBTuni-12/13) (*16*) to amplify influenza A–specific RNA (Superscript III One-Step RT PCR; Thermo Fisher Scientific Inc., Waltham, MA, USA). We fragmented amplicons and tagged them with sequencing adapters by using the Nextera XT DNA Library Kit (Illumina, San Diego, CA, USA) and run on an Illumina MiSeq sequencing system. Due to insufficient coverage of the polymerase basic (PB) 1 (segment 2) gene across many samples (mean read depth in sample with lowest coverage was 0.42×), we excluded this segment from the genome analysis. Mean coverage depth for all segments (excluding segment 2) was 2,800×. We generated consensus sequences on the basis of the most common nucleotide for a given position. In this study, we define majority genome as sequences containing all influenza genome segments except segment 2. Sequences were aligned to an influenza A(H3N2) isolate from Switzerland (GISAID accession nos. EPI614438–EPI1614444 and EPI680123; http://www.gisaid.org) by using Bowtie2 (*17*). We used Samtools and mpileup (*18*) to call single-nucleotide polymorphisms to the reference, and we used bcftools (*18*) and vcfutils (*19*) for further assembly.

### Additional Surveillance Samples

In addition to the outbreak samples, we also evaluated the hemagglutinin (HA) sequences of 13 influenza A(H3N2)–positive samples available from the same season that were not associated with these LTCF outbreaks. To eludicate the changes occurring in circulating community influenza lineages throughout the influenza season, the PHOL performs prospective surveillance by randomly selecting submitted samples for sequencing, originating from all possible sources including outpatient and inpatient care. The 13 samples selected for this analysis represent a subset of these samples that were analyzed directly from clinical specimens and had adequate HA sequence data. For these surveillance samples, we sequenced HA by using Sanger sequencing (*20*) by using the BigDye Terminator v3.1 Cycle Sequencing Kit and the ABI Prism 3730XL genetic analyzer (both from Applied Biosystems). We aligned consensus surveillance HA sequences to the H3N2 isolate from Switzerland by using MAFFT (*21*). All positions containing missing data were eliminated. We submitted all 76 majority genome outbreak sequences and surveillance HA sequences to GenBank (accession nos. MF806611–MF807155).

### Phylogenetic Analysis

We inferred evolutionary history by maximum likelihood analysis. We used PhyML (SeaView version 4.6.1, http://doua.prabi.fr/software/seaview) with a general time-reversible model and approximate likelihood ratio test branch support, where the tree with the greatest log likelihood was retained. We generated initial trees by using BioNJ (*22*) with optimized tree topology. We used FigTree version 1.4.2 (http://tree.bio.ed.ac.uk/) to manipulate the phylogenetic trees and to root to the outgroup influenza A(H3N2) isolate from Switzerland. The trees were drawn to scale (with scale bars noted) and branch length as a function of substitutions per site. We generated phylogenetic trees for both majority genome (including all outbreak pairs), as well as specifically the HA gene (including all outbreak pairs and the surveillance samples).

### Pairwise Distance Analysis

For all genes (majority genome and HA gene, including surveillance samples), we calculated pairwise distances within-outbreak sample pairs and between-outbreak sample pairs (random sample from each pair) by using MEGA-CC version 7.0.18 (*23*). We calculated pairwise distances as total number of single-nucleotide polymorphisms per base pair over the entire alignment. For between-outbreak pairs, we matched outbreaks to the most temporally coincident outbreak. The median and interquartile range of time between processing times of temporally coincident outbreak samples were 0 and 1 day, respectively.

## Results

**Figure 1:**
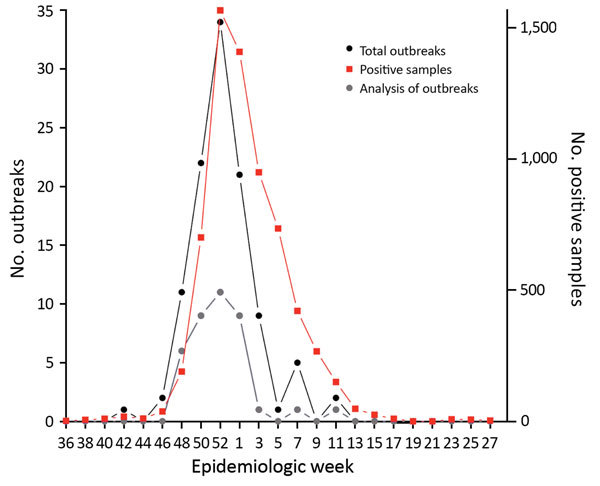
Epidemiologic curves of influenza A cases and outbreaks in long-term care facilities, by epidemiologic week, Toronto, Ontario, Canada, 2014–15. Shown are the total number (n=6,573) of influenza A–positive cases reported during the season for the province (red line), the 108 influenza A(H3N2) outbreaks in long-term care facilities analyzed at the provincial public health laboratory (black line), and the 38 outbreaks evaluated by genome sequencing in this study (gray line).

During the 2014–15 influenza season (October 15, 2014–March 23, 2015), a total of 108 influenza A(H3N2) outbreaks in Toronto healthcare institutions were laboratory-confirmed at PHOL. Of those, >2 positive H3N2 samples were confirmed in 87 outbreaks, and samples from 38 outbreaks had adequate volumes and primer amplification to be suitable for genome sequencing, and were selected for analysis. Of these analyzed outbreaks, 2 occurred in chronic care hospitals, 31 in long-term care institutions, and 5 in retirement homes. Total outbreak case counts in the analyzed outbreaks were 5–67 persons. The first outbreak analyzed occurred on November 24, 2014, and the last occurred on March 23, 2015. The temporal distribution of the 108 outbreaks, along with the 38 analyzed outbreaks and citywide positive influenza sample counts, are shown in Figure 1. The median time from recognition of a potential outbreak to the first sample processing was 1 day. The median time between the first and second samples for each outbreak pair was 0 days.

**Figure 2:**
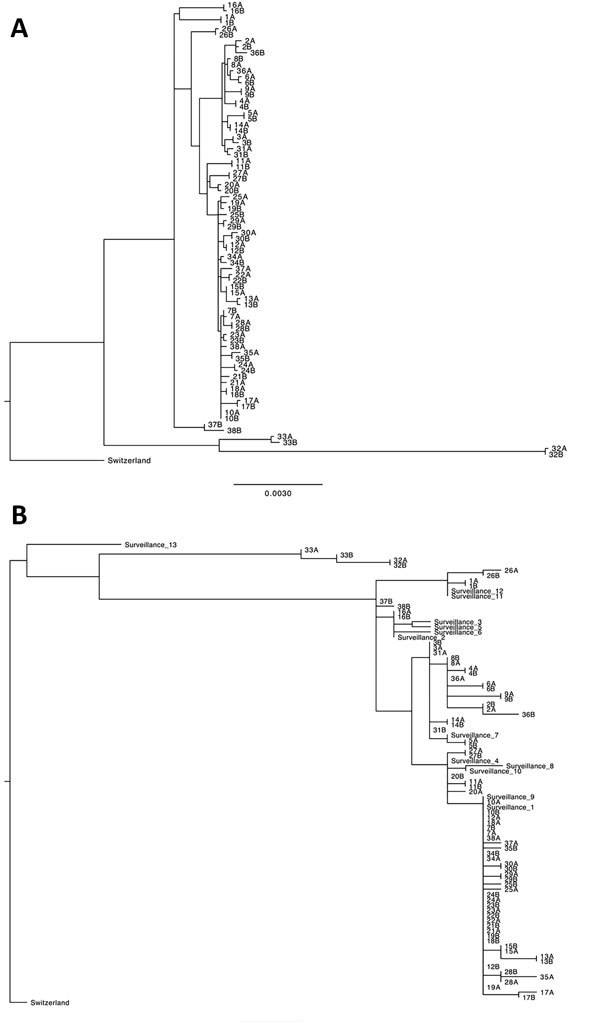
Phylogenetic trees for influenza A(H3N2) outbreak pairs from patients in long-term care facilities, Toronto, Ontario, Canada, 2014–15. A) Majority genome; B) hemagglutinin gene. Surveillance samples were included in the HA gene analysis. Scale bar indicates substitutions per site. HA, hemagglutinin.

We constructed phylogenetic trees for the majority influenza genome (Figure 2, panel A) and the HA gene alone (Figure 2, panel B). Surveillance samples with previously sequenced HA genes were included in the HA phylogenetic analysis (Figure 2, panel B).

**Figure 3:**
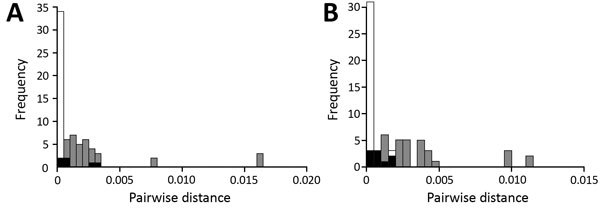
Histograms of pairwise distances for within-outbreak pairs (white) and between contemporaneous outbreak pairs (light gray) for influenza A(H3N2) samples from patients in long-term care facilities, Toronto, Ontario, Canada, 2014–15. A) Majority genome; B) hemagglutinin gene. Black indicates overlap between categories.

**Figure 4:**
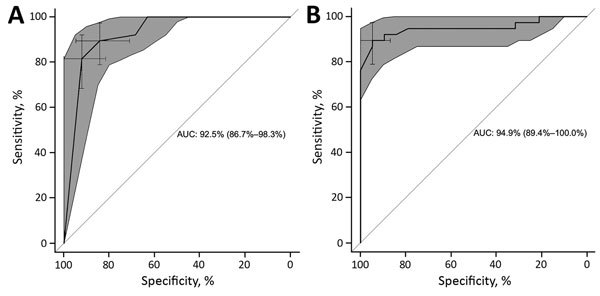
Receiver operating characteristic curves for majority genome (A) and hemagglutinin gene (B) testing for influenza A(H3N2) samples from patients in long-term care facilities, Toronto, Ontario, Canada, 2014–15. AUC values and 95% CIs are shown. The predicted binary outcome is within versus between (contemporaneous) outbreaks. AUC, area under the curve.

Histograms of pairwise distances for within-outbreak pairs and between contemporaneous outbreaks are shown in Figure 3. Pairwise distances for majority genomes ranged 0–0.003 for pairs within outbreaks and 0.0002–0.016 for pairs between outbreaks. For the HA gene, pairwise distances ranged 0–0.0018 for pairs within outbreaks, and 0–0.011 for pairs between outbreaks. A receiver operator characteristic curve (ROC) analysis (Figure 4) for differentiating within- and between-outbreak pairs identified an optimal cutoff for pairwise distances in majority genome analysis of 0.0005, giving an area under the curve (AUC) of 0.95 (95% CI 0.89–1.00) and sensitivity and specificity of 0.89 and 0.95, respectively. Of 38 outbreak pairs, 2 demonstrated between-outbreak relatedness that was equal to or greater than within-outbreak relatedness, and both of these pairs demonstrated within-outbreak pairwise distances greater than the ROC defined threshold value (Figure 2, pairs 25 and 37). Two optimal cutoffs for pairwise distances in HA gene-specific analysis were identified, and we selected the threshold (0.0009) with the most balanced sensitivity and specificity, which provided an AUC of 0.93 (95% CI 0.87–0.98) (Figure 4) and sensitivity and specificity of 0.89 and 0.84, respectively. For HA gene analysis, 2 of 38 outbreak pairs demonstrated between-outbreak relatedness equal to or greater than within-outbreak relatedness, and both of these pairs demonstrated within-outbreak pairwise distances greater than the ROC-defined threshold value (Figure 2, pairs 25 and 37). We used a Wilcoxon rank-sum test to compare the outbreak case numbers when majority genome pairwise distances were below the optimal ROC threshold (34/38 outbreaks) and when pairwise distances were higher than the optimal ROC threshold (4/38). We found no statistically significant difference in distribution of outbreak sizes between the 2 groups (p = 0.94).

## Discussion

In this retrospective genomic study of influenza A(H3N2) outbreaks in LTCFs during the 2014–15 influenza season in Toronto, we evaluated the potential role of genome sequencing in clinically defined outbreaks with >2 available pairs of H3N2-positive respiratory specimens. Our analysis indicates that, in ≈90% of cases, the initial sample pairs were sufficiently closely related, suggesting that direct transmission had occurred. Thus, routine genome sequencing for supporting influenza outbreak definitions is unlikely to add statistically significant improvements over current clinical definitions.

Epidemiologic curves for the influenza season evaluated in this study show that activity (based on submitted samples to the provincial public health laboratory) peaked at week 52, and the number of analyzed outbreaks paralleled these trends. Outbreaks ranged widely in sizes and reflect a large patient population affected by influenza effects and outbreak mitigating measures.

To determine whether these influenza outbreaks were consistent with LTCF person-to-person spread, we constructed phylogenetic trees; the majority genome and HA gene trees show that most outbreak pairs are closely related. Including HA gene analysis of surveillance samples from the same season confirmed that the specific outbreak pairs generally appear to be sampled from the same population as the surveillance cases, which we inferred to be representative of circulating influenza lineages. This finding supports the idea that outbreak strains are being introduced from broadly circulating virus lineages as opposed to circulating preferentially within an LTCF reservoir (*9*). It is likely that the remaining 10% of cases that appeared unrelated represent transmission from occult sources. However, further study of the nature and epidemiology of these seemingly unlinked outbreaks is needed.

Although most outbreak pairs were closely related, some were not. To quantify this, we calculated a pairwise distance matrix and compared distance within pairs from the same LTCF with outbreaks occurring in a different location but at the same time. When we used the majority genome analysis, the pairwise distances within outbreaks formed a Poisson-shaped distribution abutting zero genetic distance. Distribution of pairwise distances between outbreaks that overlapped was normal but was centered to the right of the within-outbreak pairwise distances. We generated an ROC curve to assist with applying a threshold to classify those outbreak pairs that were or were not caused by direct transmission. The AUC for this curve was high, indicating a strong ability to discriminate among, within, and between outbreak pairs. Analysis of the HA pairs shows similar relationships but with less clearly differentiated pairwise distance distributions. We found no obvious classification benefit of majority genome sequencing versus HA-specific gene sequencing. This finding is supported by a report of a multisite outbreak in which strain differentiation with whole-genome sequencing offered no obvious benefit over HA/neuraminidase gene sequencing for infection control purposes (*24*).

From these analyses, we see that nearly all pairs within outbreaks appeared to be highly related, likely representing linked transmission occurring within individual LTCFs. These findings suggest that current clinically defined outbreak definitions for identifying within-LTCF transmission when >2 samples are positive for influenza are highly specific. Moreover, the high proportion of related strains reinforces the importance of measures for mitigating transmission within facilities through established and routine approaches (e.g., hand and respiratory hygiene, management of ill healthcare workers, adherence to infection control practices, vaccination) (*25*). Although we demonstrate reasonable approaches to differentiate among, within, and between outbreak strains, it is unlikely that these would be useful for prospective classification of outbreaks in the facilities evaluated in this report, considering the high incidence of presumed related strain outbreaks. This conclusion is supported by the fact that outbreak size appeared to have no obvious relationship to strain relatedness. There may, however, be utility for gene sequencing in acute-care environments to better ascertain transmission sources and support infection prevention and control investigations (*26*). Moreover, it is reasonable to expect that this approach could be applied to other subtypes of influenza A.

This study has several limitations. First, we were only able to assess outbreaks with >2 PCR-positive influenza A(H3N2) samples and cannot comment on the validity of outbreak definitions in circumstances under which only 1 patient is positive for influenza. One may expect the specificity of the clinical definition in this context to be lower, although it is challenging to validate this in the absence of microbiologic sample availability (which may be compounded by limitations of influenza tests, specifically false negatives) (*27*). Similarly, additional microbiologic samples are often not obtained after the outbreak has been initially identified, which prevents us from assessing ongoing strain relatedness throughout the evolving outbreak. Our surveillance sample was also limited, in both number of sequences and length of the alignment, impairing our ability to thoroughly characterize and compare the features of influenza circulating in the 2 settings. Because of limitations of data linkage with epidemiologic data, we cannot assess other features of outbreaks, including LTCF size and geographic location, as well as individual patient factors and epidemiologic links. However, the relative consistency of the results suggests these factors are likely less relevant. Last, we did not seek to evaluate the role of deep sequencing in ascertaining linked transmission, although this particular approach warrants additional study (*28*).

In summary, current clinical definitions of influenza A outbreaks with >2 positive influenza samples appear reasonably specific for identifying presumed within-facility transmission. As a result, routine gene sequencing as part of outbreak identification does not offer clear additional benefit. However, whole/majority genome or HA-specific sequencing may prove useful to identify sources of influenza introductions where it is clinically indicated.
